# Analyzing the impact of non-participation in the FIFA World Cup Qatar 2022 on LaLiga players' physical performance

**DOI:** 10.3389/fspor.2024.1385267

**Published:** 2024-04-05

**Authors:** Gonzalo Reverte-Pagola, Javier Pecci, Juan José del Ojo-López, Roberto López del Campo, Ricardo Resta, Adrián Feria-Madueño

**Affiliations:** ^1^ Department of Physical Education and Sport, University of Seville, Seville, Spain; ^2^ Physical Coach, Sevilla Football Club, Seville, Spain; ^3^ Department of Competitions and Mediacoach, LaLiga, Madrid, Spain

**Keywords:** soccer, football, match analysis, time-motion analysis, GPS device

## Abstract

**Background:**

Monitoring external load demands in soccer is crucial for optimizing performance and reducing injury risk. However, events like the FIFA World Cup Qatar 2022 and unexpected interruptions can disrupt load management strategies. Understanding the impact of such events on player performance is essential for effective training and recovery strategies.

**Objective:**

This study retrospectively assessed the impact of the FIFA World Cup Qatar 2022 on the physical performance of LaLiga elite soccer players who were not part of the tournament. The aim was to analyze various external load parameters and determine the direction of their changes post-tournament.

**Methods:**

Data from 239 LaLiga players who were not selected for the World Cup were analyzed. External load parameters from 8 matches before and after the tournament were compared. Statistical analyses, including repeated measures ANOVA, were conducted to evaluate changes in performance metrics.

**Results:**

Minutes played and total distance covered showed no significant changes post-tournament. However, maximal speed decreased significantly (*p* < 0.001; *η*^2^*_p_* = 0.117). High-speed running parameters improved significantly (*p* < 0.05), except for HSRRelCount (*p* = 0.074; *η*^2^*_p_* = 0.013). Sprint-related variables demonstrated significant enhancements, except for SprintAbsAvgDuration, SprintMaxAvgDuration, and Sprints >85% Vel Max. Acceleration metrics showed significant improvements in Accel_HighIntensityAccAbsCount (*p* = 0.024; *η*^2^*_p_* = 0.021), while Accel_Accelerations showed no significant changes. Deceleration metrics remained unchanged, but Accel_HighIntensityDecAbsCount and Accel_HighIntensityDecAbsDistance increased significantly post-tournament (*p* = 0.002; *η*^2^*_p_* = 0.040, *p* = 0.001; *η*^2^*_p_* = 0.044, respectively).

**Conclusion:**

Non-participant LaLiga players demonstrated enhanced performance in most external load metrics after the FIFA World Cup Qatar 2022. These findings highlight the importance of effective load management during periods of competition interruption and suggest strategies to optimize performance and reduce injury risk. Further research should consider holistic performance metrics and internal load parameters to provide comprehensive insights into player response to mid-season tournaments.

## Introduction

Monitoring external load demands of the game is a challenge for team sports such as soccer (1, 2). Weekly changes in external load are necessary to optimize the microcycle in order to obtain the maximum performance during games and to reduce injury risk (3). Nonetheless, the monitorization of the external loads and thus the demands of the game constitutes the central element from which the microcycle is organized (4, 5). In this context, it is important to note that the physical performance of the soccer players Typically experience seasonal fluctuations that should be monitorized to orient training loads and thus regulating the training stimulus (6). Therefore, soccer players performance during games are strongly influenced by the moment of the season. Consequently, several studies have examined the parameters of external load across a typical season (7, 8). Several forms of assessing external load have emerged during recent years such as the use of global navigation satellite systems (GNSS) (9), local positioning systems for indoor competitions (10) or tracking systems through high-definition cameras (11). All these methods have shown to be reliable and valid for assessing external load demands of the game (12), which is an essential task for designing microcycles and training sessions (13, 14). Nonetheless, certain events, such as the interruption of the competition due to COVID-19 (15), the recent introduction of a mid-season World Cup (16, 17), or breaks for international competitions without season-to-season patterns (18), can disrupt the distribution of loads during the season. However, professional soccer teams need information about the impact of these emerging events to better understand the implications in physical performance, especially in the main external load parameters such as total distance covered, accelerations, decelerations, high-speed running or sprinting, and thus manage the load patterns. FIFA World Cup Qatar 2022 constituted a challenging event for soccer teams, since the regular competition was interrupted for a month (17, 19). This tournament created a scenario unprecedented until now, where some players competed at the highest competitive level for a month, while others would not receive a competitive stimulus for over 4 weeks. While it is known that similar precedent scenarios such as COVID-19 lockdown resulted in a decrease in match external load outcomes performance (15), it is unknown how shorter breaks such as FIFA World Cup Qatar 2022 affected to the Spanish professional teams. If training loads are not well managed, this period could partially detrain World Cup non-participants. Short detraining periods of 2–4 weeks have demonstrated to not affect maximal neuromuscular responses in soccer players (20, 21). However, they could have a negative impact on repeated sprint ability (RSA) (22, 23) or body composition (24). Nonetheless, to the best of our knowledge, no previous studies have analyzed the impact of mid-season tournaments such as the FIFA World Cup Qatar 2022 on non-participants that reduce the competition but not the training stimulus. Some authors (17) advanced that this tournament would affect to the physical performance of the players, but without clear statements about the directions of the changes suffered due to the World Cup.

Assessing the impact of mid-season events such as FIFA World Cup Qatar 2022 could help strength and conditioning coaches to optimize the training process during periods of lack of competition. Specifically, knowing the variables that are most affected after a period of competitive inactivity can help create training tasks that promote the execution of those actions with greater impact, thus improving external load management. Consequently, the aim of the present study was to retrospectively assess the impact of the FIFA World Cup Qatar 2022 on physical performance of LaLiga elite soccer players. The main hypothesis was that the period of inactivity has affected the external load metrics performance. Nonetheless, this study could determine in which direction (i.e., improvement or deterioration) the variables were affected.

## Materials and methods

### Study design

The study employed a retrospective design to evaluate how the performance of LaLiga players who were not called up by their national teams was influenced by the FIFA World Cup Qatar 2022. It compared the external load parameters observed in the 8 matches leading up to the World Cup with those recorded in the 8 subsequent matches during the regular league season.

### Sample

Data were collected from professional soccer players from LaLiga who were not selected to represent their national teams in the FIFA World Cup Qatar 2022. Specifically, data were retrospectively collected from the 8 matches played by each player before the World Cup and 8 matches following the resumption of domestic league competition. For inclusion in the analysis, players were required to have accumulated a minimum of 90 min of playtime across the 8 pre-World Cup matches and 90 min across the 8 post-World Cup matches. Moreover, no injured players during data collection period were included in analyses. In total, 239 players met these criteria and were included in the analyses. There were included 98 defenders, 100 midfielders and 41 forwards with 27.39 ± 4.18 years old.

### Variables

In the assessment of external load parameters, the study gathered the following variables for each player in every match: minutes of game played, total distance covered in meters, maximal speed attained, high-speed running (HSR) outcomes, sprint outcomes, acceleration outcomes, and deceleration outcomes, which have been established as key performance indicators and key external load metrics to define the player profile (25–27).

HSR variables scrutinized encompassed the total count of high-speed running actions (>21 km/h) (HSRAbsCount), the total distance covered during high-speed running (HSRAbsDistance), the total duration of high-speed running (HSRAbsDuration), the number of actions performed at speeds exceeding 75.5% of the player's historical maximum speed based on the WIMU (i.e., GPS) Profile (HSRRelCount).

Sprint variables examined included total time spent sprinting over 24 km/h (SprintDur), total count of sprinting actions over 24 km/h (SprintAbs), average duration of sprinting actions (SprintAbsAvgDuration), total duration of all sprinting actions (SprintAbsDuration), count of the number of times a player has repeated an absolute sprint during a predefined RSA period of 60 s (SprintAbsRepetitions), count of sprints exceeding 85% of the player's maximal velocity based on the WIMU profile (Sprints >85% Vel Max), overall distance covered during sprints (PlayerDistanceSprint), and total count of sprints performed (PhysicalNumberofSprints).

Acceleration outcomes comprised the count of all accelerations over 3 m/s^2^ (Accel_Accelerations), and total count of high-intensity accelerations (Accel_HighIntensityAccAbsCount). Lastly, deceleration outcomes entailed the count of all decelerations (Accel_Decelerations), total count of high-intensity decelerations under −3 m/s^2^ (Accel_HighIntensityDecAbsCount), and total distance covered during high-intensity decelerations under −3 m/s^2^ (Accel_HighIntensityDecAbsDistance).

### Instruments

Following previous data collection described methodology (28), running performance during matches was evaluated using an advanced multicamera computerized optical tracking system called TRACAB (ChryronHego VID, New York, NY). This system was managed through the Mediacoach application (LaLiga, Madrid, Spain), operating at a sampling frequency of 25 Hz (i.e., 25 samples per s). Previous studies assessing the reliability and accuracy of this system for the designated variables demonstrated strong correlations (*r* > .80) and high intraclass correlation coefficients (*r* > .75) between the Mediacoach multicamera tracking system and the Global Positioning System. Additionally, minimal standard errors of estimate (≤.60) were observed across all speed categories analyzed in this investigation (11, 29, 30).

### Statistical analysis

Three distinct analyses were performed to assess how the FIFA World Cup Qatar 2022 affected player performance, with each analysis focusing on specific variables. Initially, alterations in players’ minutes of game played were explored by summing the minutes from the 8 matches before and after the World Cup. A repeated measures analysis of variance (ANOVA) was then applied to compare the differences in playing time pre and post-tournament. Secondly, the Maximal Speed variable was assessed by identifying the highest speed attained by players in matches lasting over 60 min before and after the World Cup. Subsequently, a repeated measures ANOVA was used to assess variations in maximal speed pre and post-tournament. Finally, mean values for the remaining performance variables (total distance covered, HSR, sprints, accelerations, and decelerations) were calculated for matches played before and after the World Cup, considering only those where players participated for over 60 min. Repeated measures ANOVA tests were conducted to compare the means of each variable before and after the World Cup, offering insight into the effects of the nearly month-long interruption without competitions on performance aspects of players who were not selected for the World Cup. All statistical analyses were performed using Jamovi Statistical Software (The jamovi project (2022). jamovi. (Version 2.3) [Computer Software]. Retrieved from https://www.jamovi.org) and Microsoft Excel. The significance level for the statistical tests was set at *p* < 0.05 with a 95% confidence interval (CI). Effect sizes were also calculated using partial eta squared (*η*^2^*_p_*) to assess the magnitude of change in the variables, with values of 0.01 considered small, 0.06 medium, and over 0.14 large.

## Results

### Impact of FIFA World Cup Qatar 2022 on minutes of game

**Figure 1:**
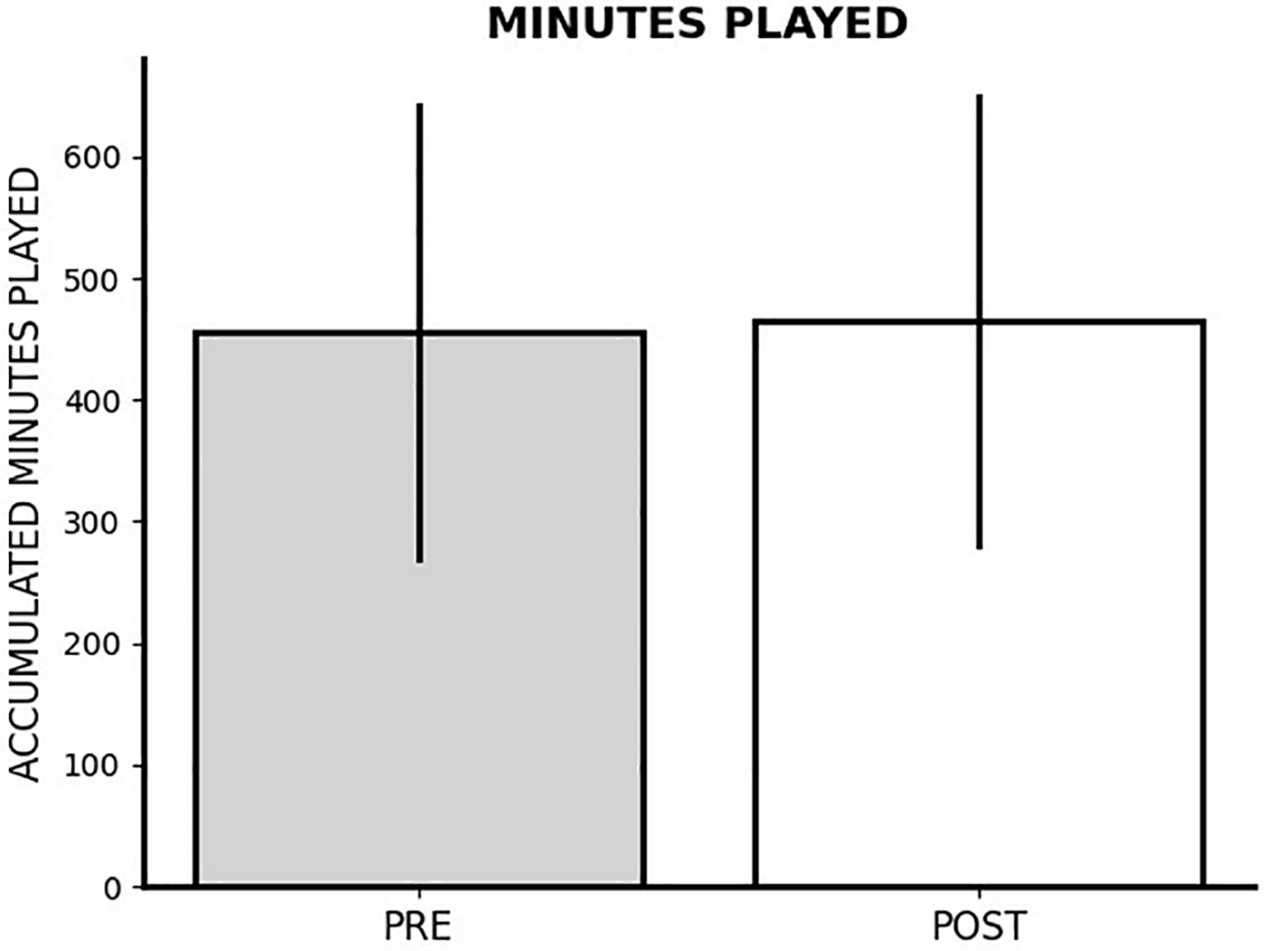
Changes in accumulated minutes played after FIFA World Cup Qatar 2022.

The repeated measures ANOVA test indicated a non-significant effect on minutes of game played (*p* = 0.468; *η*^2^*_p_* = 0.002) when comparing pre-World Cup vs. post-tournament periods. Similarly, there was a non-significant difference in the number of matches with >60 min played before and after the World Cup (*p* = 0.109; *η*^2^*_p_* = 0.011). Figure 1 provides a summary of the alterations in minutes played.

### Impact of FIFA World Cup Qatar 2022 on total distance

**Figure 2:**
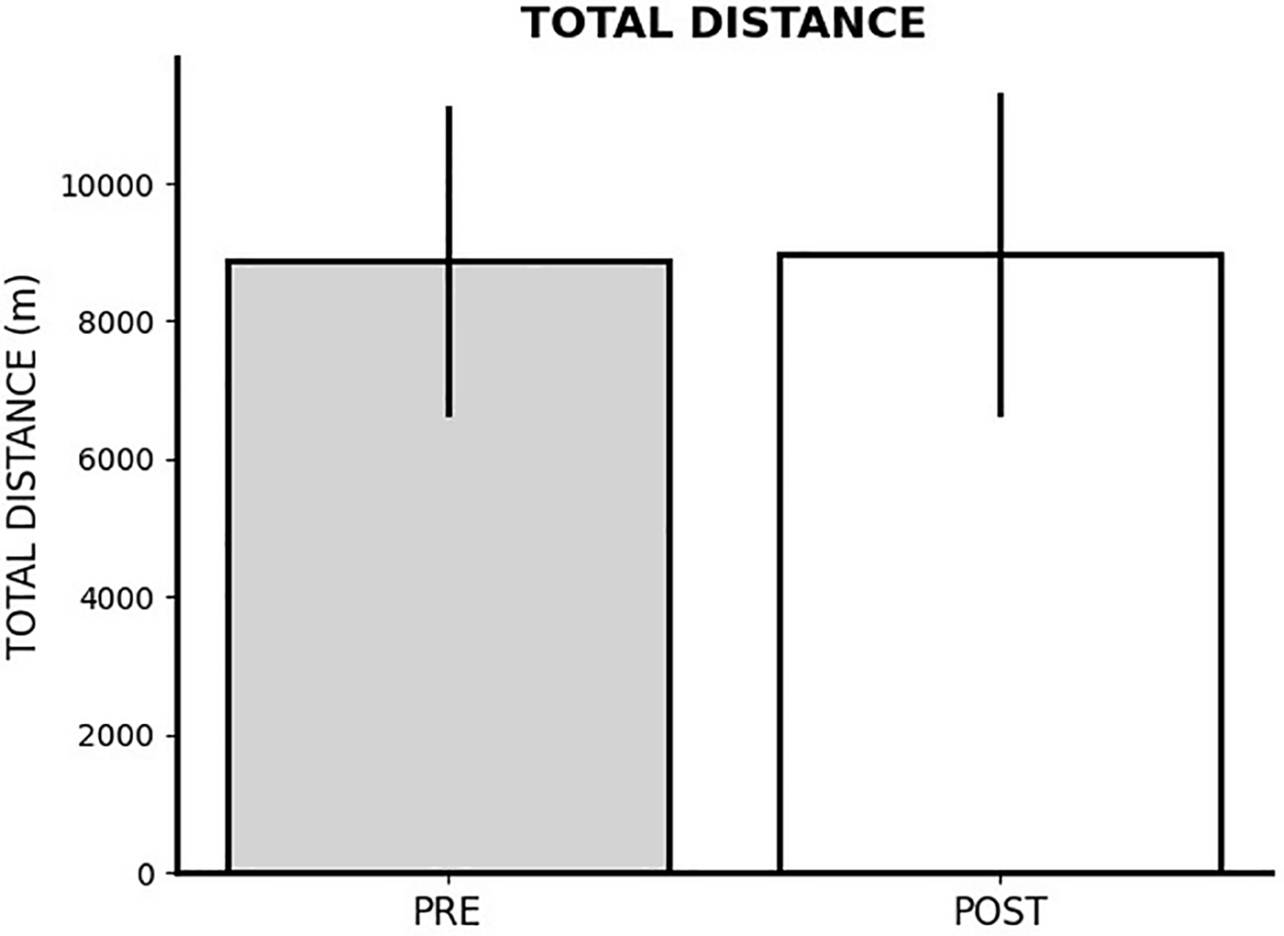
Changes in total distance covered after FIFA World Cup Qatar 2022.

The repeated measures ANOVA test revealed non-significant differences between pre- World Cup vs. 8 subsequent matches in PlayerPhysicalDistance (*p* = 0.407; *η*^2^*_p_* = 0.003). Figure 2 shows changes in total distance covered.

### Impact of FIFA World Cup Qatar 2022 on maximal speed

**Figure 3:**
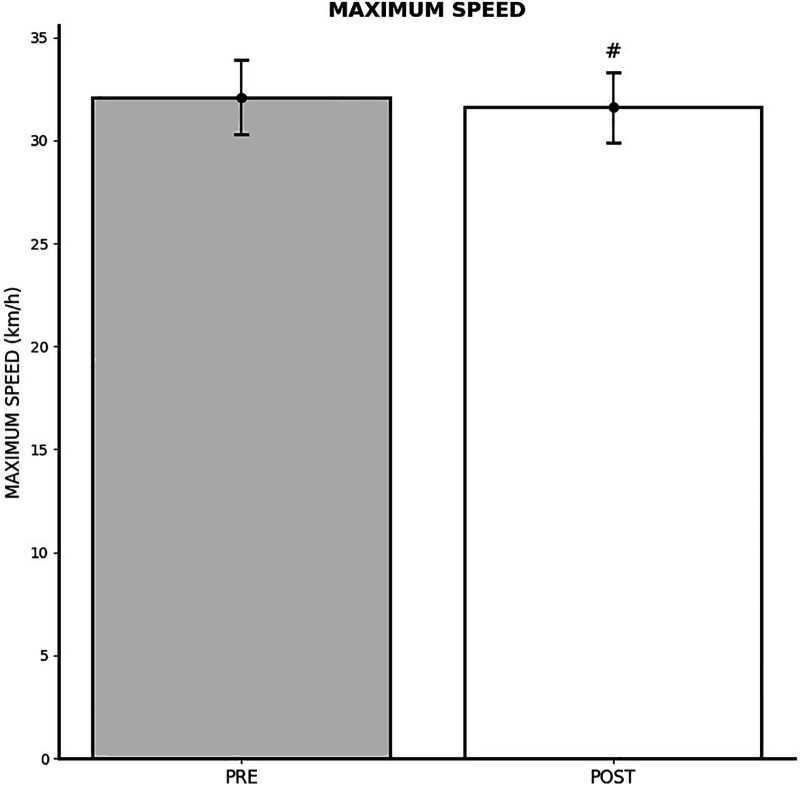
Changes in maximum speed reached after FIFA World Cup Qatar 2022. ^#^Significantly lower than PRE.

The repeated measures ANOVA test indicated a significant influence of the World Cup on PlayerPhysicalMaximumSpeed (*p* < 0.001; *η*^2^*_p_* = 0.117), suggesting the superiority of pre-tournament outcomes. Maximal speed changes are shown on Figure 3.

### Impact of FIFA World Cup Qatar 2022 on high-speed running outcomes

**Figure 4:**
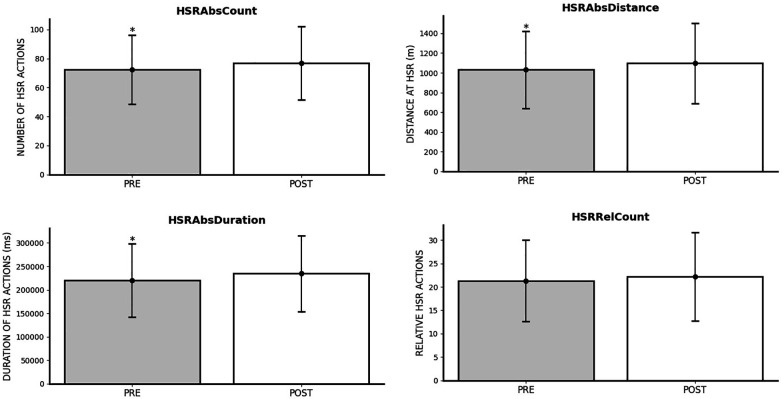
Changes in high-speed running outcomes after FIFA World Cup Qatar 2022. *Significantly lower than POST.

The repeated measures ANOVA test demonstrated significant differences when comparing 8 previous FIFA World Cup Qatar 2022 matches vs. 8 subsequent matches on various high-speed running parameters. Specifically, HSRAbsCount (*p* < 0.001; *η*^2^*_p_* = 0.048), HSRAbsDistance (*p* = 0.001; *η*^2^*_p_* = 0.043), and HSRAbsDuration (*p* < 0.001; *η*^2^*_p_* = 0.051) all showed the superiority of post-tournament outcomes. However, HSRRelCount (*p* = 0.074; *η*^2^*_p_* = 0.013) exhibited non-significant effects of time measures. High-speed running outcomes differences pre-post World Cup are shown on Figure 4.

### Impact of FIFA World Cup Qatar 2022 on sprint outcomes

**Figure 5:**
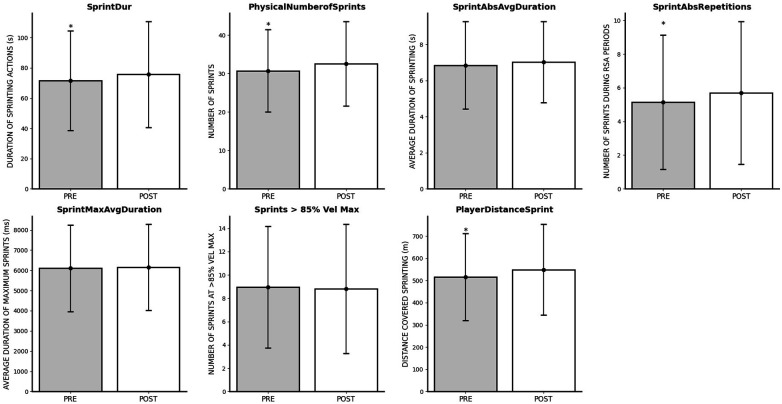
Changes in sprinting outcomes after FIFA World Cup Qatar 2022. *Significantly lower than POST.

The repeated measures ANOVA test revealed significant differences between time points across various sprint-related variables. Specifically, PlayerDistanceSprint (*p* = 0.001; *η*^2^*_p_* = 0.042), PhysicalNumberofSprints (*p* = 0.001; *η*^2^*_p_* = 0.042), SprintDur (*p* = 0.010; *η*^2^*_p_* = 0.028), SprintAbs (*p* = 0.004; *η*^2^*_p_* = 0.034), SprintAbsDuration (*p* = 0.003; *η*^2^*_p_* = 0.038), SprintAbsRepetitions (*p* = 0.015; *η*^2^*_p_* = 0.026) all favored post-World Cup outcomes. However, SprintAbsAvgDuration (*p* = 0.306; *η*^2^*_p_* = 0.004), SprintMaxAvgDuration (*p* = 0.780; *η*^2^*_p_* < 0.001) and Sprints >85% Vel Max (*p* = 0.658; *η*^2^*_p_* = 0.001) exhibited non-significant effects of time measures. An overview of the changes in sprinting parameters is shown in Figure 5.

### Impact of FIFA World Cup Qatar 2022 on accelerations outcomes

**Figure 6:**
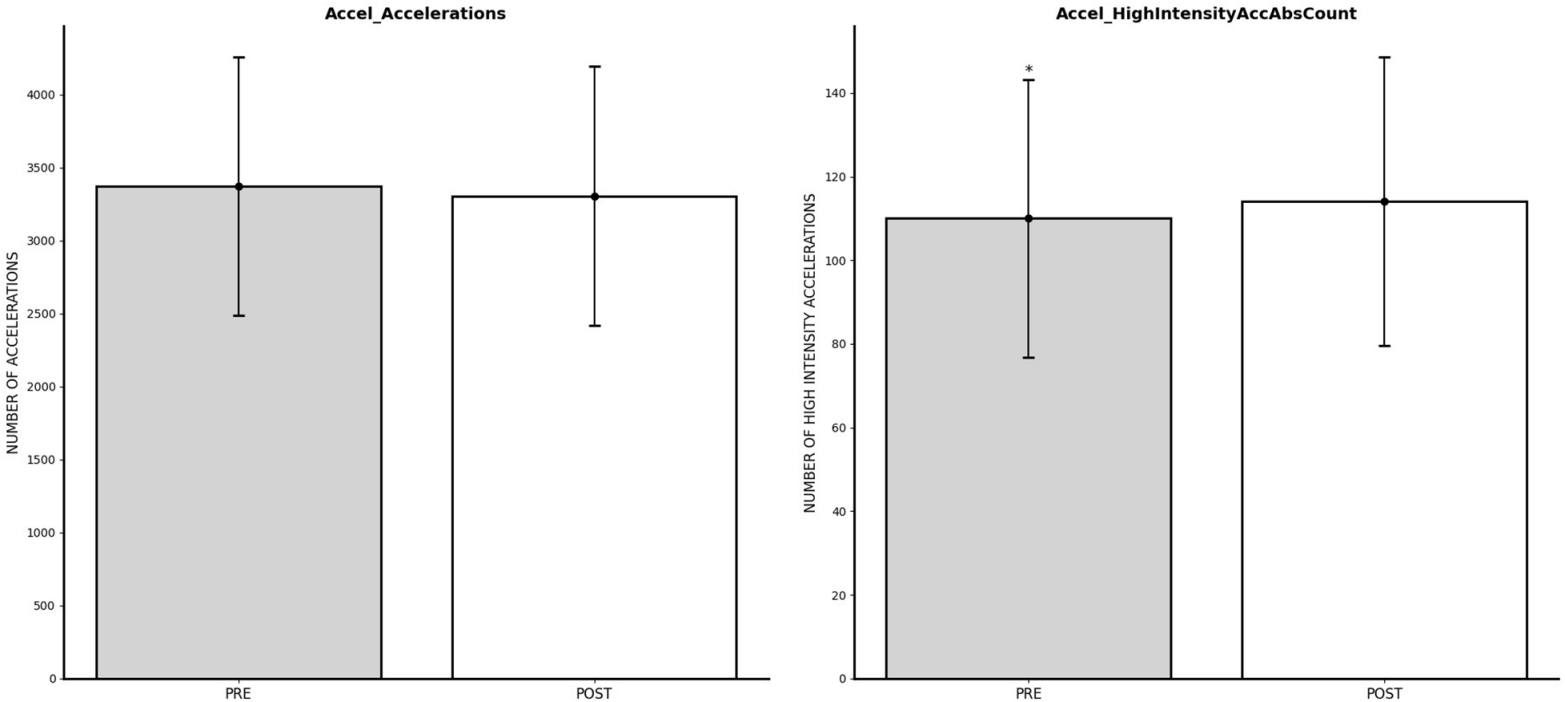
Changes in acceleration outcomes after FIFA World Cup Qatar 2022. *Significantly lower than POST.

The repeated measures ANOVA test indicated significant effects of the World Cup on Accel_HighIntensityAccAbsCount (*p* = 0.024; *η*^2^*_p_* = 0.021), demonstrating significant improvements in post-World Cup measures. However, Accel_Accelerations (*p* = 0.193; *η*^2^*_p_* = 0.007) exhibited non-significant effects of time measures. Acceleration changes are shown on Figure 6.

### Impact of FIFA World Cup Qatar 2022 on deceleration outcomes

**Figure 7:**
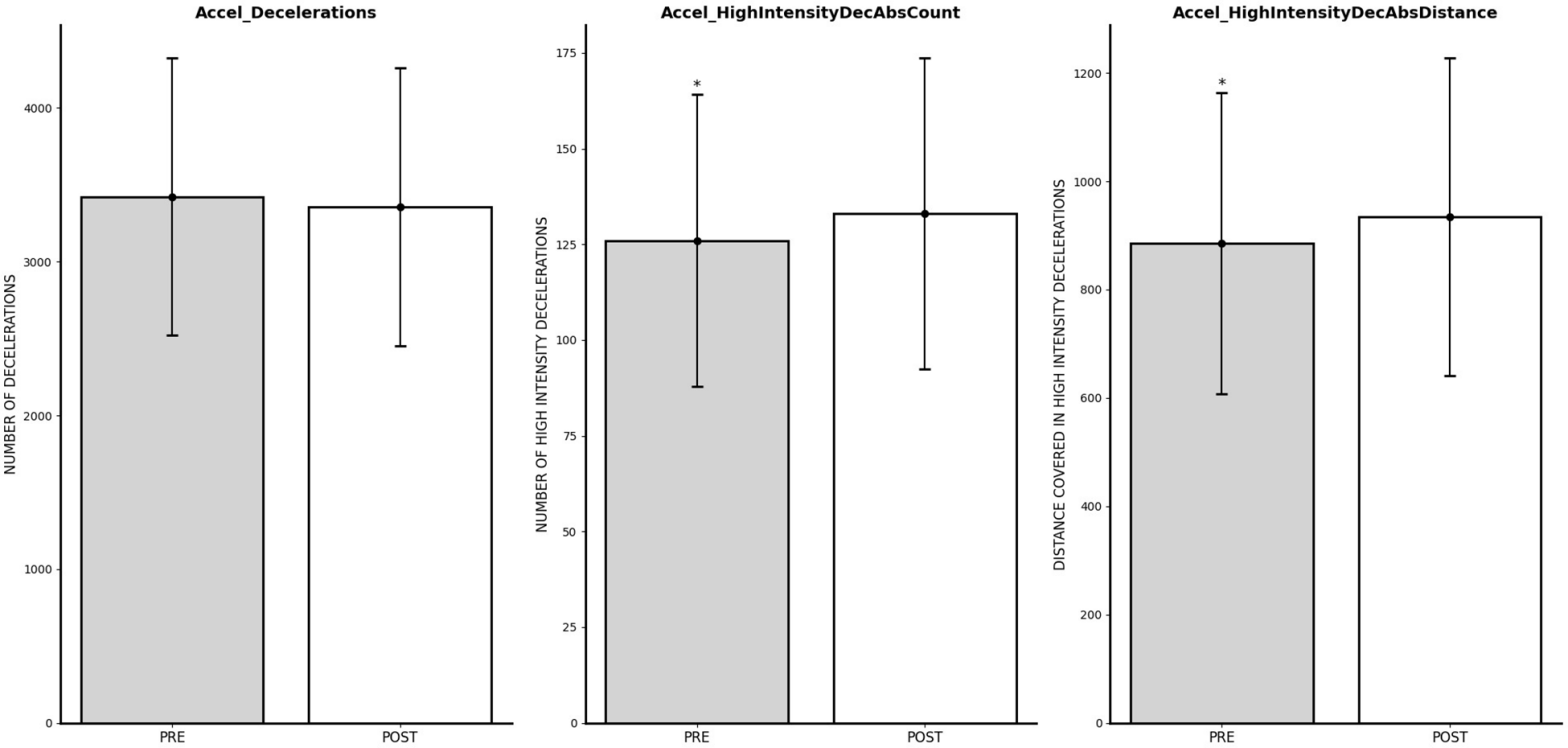
Changes in deceleration outcomes after FIFA World Cup Qatar 2022. *Significantly lower than POST.

The repeated measures ANOVA test revealed no significant differences between pre-World Cup vs. post-World Cup periods in Accel_Decelerations (*p* = 0.191; *η*^2^*_p_* = 0.007), but significant increases after World Cup on Accel_HighIntensityDecAbsCount (*p* = 0.002; *η*^2^*_p_* = 0.040), and Accel_HighIntensityDecAbsDistance (*p* = 0.001; *η*^2^*_p_* = 0.044), indicating significant enhancements in post-World Cup measures. Pre-post World Cup differences are displayed on Figure 7.

## Discussion

The present study aimed to assess the impact of FIFA World Cup Qatar 2022 on non-participants physical performance in LaLiga players analyzing 8 matches pre- and post-tournament. Before this study, there were no information about the impact of the World Cup on regular season performance variables and our results could help coaches to better manage future similar events. According to our initial hypothesis, this study showed substantial changes in match performance in most of the analyzed variables, revealing significant increases in most of the analyzed variables, except for maximal speed. Nonetheless, maximum speed performance showed a significant reduction after FIFA World Cup Qatar 2022. These findings could help coaches to emphasize the training of this variable. It could be of special relevance the achievement of similar or even greater maximum speed of sprinting, since sprinting is the most frequent action prior scoring a goal (31). In addition, a review has highlighted the importance of this action in the game given the development of the game and due to the increasing peak velocities observed in soccer players (32). Therefore, disposing a higher sprinting speed could determine the difference between scoring or not a goal. This reduction in maximal speed could be attributed to the lower number of maximal sprinting scenarios during training when compared to official matches. In this line, the cessation of competition could have resulted in a reduction of the performance in maximal speed. Therefore, during periods of inactivity of competition maximal sprinting speed tasks should be carried out to not decrease performance when returning to competition. In addition, this reduction could be also explained by the decrease in the number of minutes played when comparing pre-post World Cup and thus in the exposure of competitive stimulus. In addition, our analyses showed a medium effect size for the changes produced in maximal speed, reinforcing the need for focusing on this training variable during short breaks of competition. The number of high intensity accelerations using absolute thresholds showed a significant increase when comparing pre-post World Cup values. Nonetheless, it is important to highlight the importance of contextualizing the thresholds as showed in previous studies (33, 34), but it is also important to note that no consensus exists about the implementation of specific and relative thresholds (34), so future studies should reach consensus about the implementation of relative thresholds to further analyzing changes in high-intensity accelerations after mid-season tournaments such as the analyzed in the present study and to report these values in a better way. Anyway, the number of high intensity accelerations has shown to be the greatest during matches in the microcycle pattern (i.e., match demands in number of accelerations are greater than in training sessions) (35, 36). Nevertheless, previous research has shown that number of accelerations decrease throughout the season (36), but the results of the present study have shown an increase in this parameter, so substantial breaks in competition could result in an increase in high-intensity accelerations and most of the external load variables based on the results of the present study. Moreover, the correlation between accelerations performed during training sessions and accelerations during matches is very low (37), so performing more accelerations during training not necessarily result in greater performance during matches, reinforcing that it is the break in the competition that has generated an increase in this metric and not a greater number of accelerations in training. However, future studies should analyze the load patterns in training sessions to conclude if the possible modification of the training periodization could have influenced these changes in match outcomes.

The main finding of the present study is that external load parameters were greater after the FIFA World Cup Qatar 2022. Results of the present study showed greater performance in variables related to high-speed running over 21 km/h, greater performance in most of the sprinting variables and greater performance in deceleration variables. The results of the present work are in line with findings that neuromuscular performance after short-term detraining could be enhanced (20, 21), Nonetheless, the detraining period in this study should be understood as a break of competitive matches, since on-field training continued in this period. To the best of our knowledge, this is the first study in assessing the differences in external load parameters after a cessation of the competition but not in training. Future studies should address the pattern of loads performed during these periods without competition to better understand the mechanism in the improvement of the external loads parameters as shown in this study. However, these findings could help coaches to better manage recovery strategies, as well as load management. In addition, it would be interesting to analyze the impact of FIFA World Cup Qatar 2022 on those players who participated in the tournament. Greater exposures to high-speed running and sprinting have demonstrated to produce high fatigue levels in the posterior chain and especially in the hamstrings (38–40), which could increase the risk of injury in this muscle group (39, 41). In this line, it could be essential to optimize the recovery strategies to minimize loading on the hamstrings and improve sport performance (42, 43). However, increasing fitness levels, as well as neuromuscular strength levels is the best strategy to enhance recovery, since players with greater fitness and strength levels have demonstrated to recover faster (44). Therefore, strength and conditioning coaches should take advantage of these periods without competition to increase fitness levels and strength performance. In addition, these results could serve to enhance team management, since based on the results of this study, coaches can anticipate better physical performance from players who have not participated in the mid-season tournament. In addition, our study provides valuable insights into how coaches can adjust training regimens to optimize player performance, particularly in areas such as sprinting speed and high-intensity accelerations, which are crucial for on-field success.

Several limitations should be highlighted from the present study. Firstly, only physical performance has been evaluated and the results of the present study should be complemented with technical-tactical metrics, because better physical performance does not necessarily translate to better sport performance (45, 46). A more holistic approach integrating technical-tactical factors could help optimize the training process by better understanding the effects of mid-season tournaments such as FIFA World Cup Qatar 2022 on different dimensions of the game. Consequently, the analysis of technical-tactical metrics could have impacted on an integrated practical application on how the training of sprinting speed is performed during soccer tasks that favor the worsened patterns observed in this study. Another limitation that should be highlighted is the lack of analysis of internal load parameters, that could add valuable information to understand how the increase in external load parameters demonstrated in this study affect to the response of the players. Integrating internal load parameters is necessary to improve the periodization of the microcycles, thus contributing to improve the implications of this study on performance and the reduction of non-contact injury risk (47) by managing properly the player load. Previous studies have shown a clear relationship between external and internal load parameters and injury risk (48), so this study could contribute to a better management of load patterns to reduce the injury risk. Therefore, a better integration of external and internal parameters could have provided more clear directions on how to manage not only the metrics of the players during tasks, but also their internal responses. Another limitation that could be considered is the number of matches selected for analysis, since there is no consensus about the number of observations pre- and post-event to analyze. In this line, our aim was to collect a wide range of matches to have firm results of the influence of the World Cup on physical performance, but consensus is needed in the number of matches selected for analysis. Lastly, only Spanish LaLiga players were analyzed. In this line, the influence of the country of competition (i.e., Spain) makes difficult to extrapolate the results of this study to another leagues, since substantial differences in external load and game patterns exist between top European leagues (49). Therefore, future studies should analyze how these mid-season tournaments affect to the players of different countries in order to study if the responses of external load metrics are dependent on the country and the style of playing.

## Conclusion

The present study provides valuable insights into the impact of the FIFA World Cup Qatar 2022 on the physical performance of Spanish LaLiga players who did not participate in the tournament. Our findings indicate significant improvements in various external load parameters following the World Cup, suggesting potential implications for load management and performance optimization strategies. Specifically, non-participants demonstrated enhancements in the number of high-speed running actions, distance covered at high-speed running, duration of high-speed running actions, duration of sprinting actions, number of sprints performed, number of sprints performed during repeated sprint ability periods, distance covered during sprinting, number of high-intensity accelerations, number of high-intensity decelerations, and the distance covered during high-intensity decelerations. However, it's worth noting that maximum speed performance experienced a reduction post-tournament.

These findings underscore the importance of effectively managing short periods without competition to maximize on-field performance and minimize injury risk. Coaches and sports scientists can use this information to tailor training regimens and recovery strategies during breaks in the competition calendar, thereby optimizing player readiness and overall team performance. These results have significant implications for athlete preparation and injury prevention strategies in professional soccer.

## Data Availability

The original contributions presented in the study are included in the article/supplementary materials, further inquiries can be directed to the corresponding author.
